# Serological and genetic markers in gastric polyps: Diagnostic and prognostic roles of pepsinogen, gastrin-17, and ABO antigens

**DOI:** 10.1097/MD.0000000000046964

**Published:** 2026-01-23

**Authors:** Qian Gao, Xing He, Haiqing Li, Hezhong Yan, Lili Wu, Pei Zhong, Jun Tang

**Affiliations:** a Department of Gastroenterology, The 901th Hospital of Joint Logistics Support Force, Hefei, Anhui, China; b Department of Clinical Laboratory Medicine, The 901th Hospital of Joint Logistics Support Force, Hefei, Anhui, China; c Department of Clinical Laboratory Medicine, The Second Hospital of Anhui Medical University, Hefei, China.

**Keywords:** blood type, gastric polyp, gastric-17, PGI/PGII, prediction, stomach cancer risk

## Abstract

Predictive value of pepsinogen (PG), gastrin-17 (G-17), and blood groups for differentiating solitary/multiple gastric polyps (GPs) and their size. A retrospective analysis was performed on patients with GPs confirmed by gastroscopy admitted to the Department of Gastroenterology at the 901 Hospital of the Joint Logistic Support Force of the People’s Liberation Army from December 2021 to May 2024. This included variables such as medical record number, sex, age, date of admission, primary diagnosis, and serum markers G-17, PGI, PGII, alpha-fetoprotein, carcinoembryonic antigen, and carbohydrate antigen 19-9, as well as blood type. A total of 142 subjects underwent gastroscopy, with an overall age of 55.94 ± 11.34 years and 91 females (63.6%). The ages of group A, B, O, and AB were 57.59 ± 11.21, 55.43 ± 11.77, 55.44 ± 11.91 and 53.79 ± 8.94, respectively. Gender (female) was 34 (77.3%), 19 (54.3%), 29 (58.0%), and 9 (64.3%), respectively. Correlation analysis showed that no variable was associated with blood type among all variables in the data. However, after adjusting for sex and age, blood type was found to be correlated with polyp size, G-17, pepsinogen I (PGI), pepsinogen II (PGII), alpha-fetoprotein, which was almost consistent with the results after adjusting for sex, age, hypertension, diabetes, and coronary heart disease. Finally, the area under the curve (AUC) of receiver operating characteristic (ROC) curve for age, sex G-17, PGI/PGII, and blood group predicting pathology (polyp/adenoma) was 0.943; The AUC of the ROC curve for single/multiple prediction was 0.663. The AUC of the ROC curve for the predicted size (>1 cm) is 0.820. This study found that the use of age, sex, PGI/PGII, G-17 in combination with different blood groups has important value in the identification of clinicopathological features of GPs, the relationship between single/multiple and polyp size and prediction.

## 1. Introduction

Gastric polyps (GPs) is a lumen lesion that originates above the mucosal surface.^[1]^ This concise definition encompasses a broad spectrum of lesions with distinct histological features and varying neoplastic potential. The detection of GPs has become increasingly prevalent in clinical practice, with current estimates indicating their occurrence ranged from 0.30% to 6.8% of upper gastrointestinal endoscopies performed all over the world.^[2]^ This epidemiological trend likely underrepresents the true prevalence, given that the majority of GPs remain asymptomatic throughout their clinical course.

Endoscopic surveillance may be indicated in select clinical scenarios. Most GPs demonstrate characteristic endoscopic morphology within the gastric lumen and may exhibit associations with pathological conditions such as *Helicobacter pylori* infection, autoimmune gastritis, or hereditary polyposis syndromes.^[3]^ However, histopathological evaluation of both the polypoid lesions and adjacent mucosa remains mandatory for precise clinicopathological assessment and definitive diagnosis.^[4]^ While the majority of polyps are nonneoplastic and require no intervention, certain GPs harbor malignant transformation potential or may present as overt malignancy at the time of endoscopic detection.^[5]^ Consequently, comprehensive understanding of GP’s diagnostic evaluation, risk stratification, histological classification, and clinical management proves critical for accurate prognostic determination in affected patients.

The development of a noninvasive diagnostic modality capable of reliably identifying individuals with gastric polyps holds critical importance for enhancing early detection of suspected gastric malignancies. To circumvent repetitive endoscopic procedures and improve patient compliance with surveillance protocols, several investigative strategies have been developed. Among these, serological biomarkers have garnered increasing interest for evaluating gastric mucosal atrophy.^[6]^ Multiple potential serum biomarkers, including pepsinogen I and II (PGI and PGII respectively), gastrin-17 (G-17), and *H. pylori* IgG antibodies, have been validated either individually or in combination for predicting gastric mucosal status.^[6]^ Notably, PGI is exclusively secreted by chief cells in the gastric corpus, while PGII originates from pyloric glands and proximal duodenal mucosa, with G-17 being specifically produced by G-cells in the antral mucosa.^[7]^ Several clinical studies have evaluated the diagnostic efficacy of this serological panel for noninvasive GP detection, demonstrating promising preliminary results^[8,9]^; however, conflicting evidence from other investigations challenges its clinical utility.^[10,11]^ Furthermore, emerging research indicates potential associations between ABO blood group phenotypes and various gastrointestinal pathologies, though their correlation with gastric polyps remains scarcely investigated in current literature.^[12-14]^

The classification and clinical management of GPs present considerable challenges for clinicians due to uncertainties surrounding lesion characteristics, biopsy protocols, therapeutic necessity, treatment modalities, and long-term surveillance regimens. Paramount to this process is the imperative to avoid underestimating lesions with malignant potential and to implement timely therapeutic interventions when indicated. Notably, China’s status as a high-incidence region for gastric carcinoma^[15]^ necessitates more proactive therapeutic approaches in clinical practice. This study therefore aims to systematically evaluate the predictive capacity of pepsinogen, gastrin-17, and ABO blood group phenotypes in determining histopathological profiles, polyp multiplicity (single vs multiple), and lesion dimensions among patients with gastric polyps, ultimately seeking to establish a novel predictive framework for optimized clinical decision-making.

## 2. Materials and methods

### 2.1. Study population and specimens

Consecutive patients admitted to the Department of Gastroenterology at our institution between December 2021 and May 2024, who underwent esophagogastroduodenoscopy (EGD) revealing polypoid lesions, were enrolled. Polyp tissues obtained via biopsy were preserved in 5 mL neutral buffered formalin and submitted to the Department of Pathology for histopathological analysis.

This study was conducted according to the Declaration of Helsinki and received approval from The 901th Hospital of Joint Logistics Support Force (study ID 202510001).

Inclusion criteria: Complete medical records including patient ID, sex, age, admission date, and primary diagnosis (patients with gastric polyps); age 25 to 80 years.
Exclusion criteria: History of prior total gastrectomy, contraindications to EGD, or incomplete medical records (missing ID, sex, age, admission date, or diagnosis).

All participants provided written informed consent. Diagnostic and therapeutic procedures adhered to institutional safety protocols. Demographic and clinical data were collected strictly for research purposes.

### 2.2. ABO blood group typing

Fasting venous blood samples were collected in the morning. ABO/RhD blood groups were determined using a microcolumn agglutination technique (Blood Group Antigen Test Cards, Bio-Rad Laboratories, Hercules).

### 2.3. Quantification of G-17, PGI, PGII, AFP, CEA, and CA199

Serum samples were centrifuged at 3500 rpm (relative centrifugal force 2500×g) for 10 minutes. Supernatant levels of gastrin-17 (G-17), pepsinogen I (PGI), pepsinogen II (PGII), alpha-fetoprotein (AFP), carcinoembryonic antigen (CEA), and carbohydrate antigen 19-9 (CA199) were measured via enzyme-linked immunosorbent assay (ELISA; Shanghai Jingkang Biological Technology Co., Ltd.; kits provided by Hebei Bohai Bioengineering Co., Ltd. China).

### 2.4. Statistical analysis

Data were analyzed using SPSS 17.0 (IBM SPSS Statistics, Armonk). Categorical variables were compared via Chi-square (*χ*²) tests. Normally distributed continuous variables were assessed using Student *t* test or *Q*-test, while non-normally distributed variables were analyzed with the Mann–Whitney *U* test. Nonlinear correlation plots were generated using least squares algorithm fitting in GraphPad Prism 9.1 (GraphPad Software, San Diego). A 2-tailed *P*-value < .05 was deemed statistically significant.

## 3. Results

### 3.1. Demographic characteristics

Finally, a total of 142 subjects were diagnosed with GPs after underwent endoscopic evaluation (Supplementary Figure, Supplemental Digital Content, https://links.lww.com/MD/R193), with a mean age of 55.94 ± 11.34 years. The cohort comprised 91 females (63.6%). Age distributions across ABO blood groups were as follows: Group A (57.59 ± 11.21 years), Group B (55.43 ± 11.77 years), Group O (55.44 ± 11.91 years), and Group AB (53.79 ± 8.94 years). Female predominance was observed in all blood groups: A (77.3%, 34/44), B (54.3%, 19/35), O (58.0%, 29/50), and AB (64.3%, 9/14). Histopathological classification revealed inflammatory polyps in 18.18% (26/142), hyperplastic/adenomatous polyps in 76.92% (110/142), and adenomas in 4.90% (7/142). Demographic details stratified by ABO blood group are summarized in Table 1. No participants were excluded due to incomplete data during the study period.

**Table 1:** Characteristics of studied participants stratified by blood type blood type groups.

	Type A	Type B	Type O	Type AB	Population	*P*-value
Age	57.59 ± 11.21	55.43 ± 11.77	55.44 ± 11.91	53.79 ± 8.94	55.94 ± 11.34	.664
Gender (women, %)	34 (77.3)	19 (54.3)	29 (58.0)	9 (64.3)	91 (63.6)	.138
Hospital stay	6.36 ± 1.78	6.11 ± 1.62	6.14 ± 2.01	6.07 ± 2.06	6.20 ± 1.84	.911
Polyp size						.780
≤0.5 cm	14 (31.8)	13 (37.1)	19 (38.0)	7 (50.0)	53 (37.1)	
0.6–1 cm	26 (59.1)	21 (60.0)	28 (56.0)	5 (35.7)	80 (55.9)	
1.1–2 cm	4 (9.1)	1 (2.9)	3 (6.0)	2 (14.3)	10 (7.0)	
Single/multiple (single, %)	20 (45.5)	11 (31.4)	17 (34.0)	3 (21.4)	51 (35.7)	.336
Pathology						.348
Inflammatory polyp	11	7	8	0	26	
Hyperplasia/adenomatous polyp	31	27	38	14	110	
Adenoma	2	1	4	0	7	
PGI	115.15 ± 93.00	129.99 ± 71.38	107.75 ± 75.03	103.19 ± 47.09	115.02 ± 77.93	.567
PGII	9.60 ± 7.93	13.87 ± 16.40	9.59 ± 6.86	7.42 ± 4.81	10.43 ± 10.30	.125
Gastrin-17	16.27 ± 19.45	11.35 ± 17.43	13.35 ± 19.58	11.40 ± 16.57	13.57 ± 18.67	.662
PGI/PGII	13.27 ± 5.83	13.28 ± 7.16	13.31 ± 6.64	16.53 ± 6.91	13.61 ± 6.57	.383
AFP	2.82 ± 1.38	3.28 ± 1.51	2.87 ± 1.18	2.84 ± 1.19	2.95 ± 1.33	.451
CEA	1.76 ± 0.85	2.44 ± 1.39	1.91 ± 1.15	1.59 ± 0.43	1.96 ± 1.11	.025*
CA199	9.24 ± 10.31	8.24 ± 9.28	9.63 ± 8.60	9.50 ± 6.15	9.17 ± 9.01	.942
Hypertension (incidence, %)	8 (18.2)	8 (22.9)	20 (40.0)	4 (28.6)	40 (28.0)	.107
Diabetes mellitus (incidence, %),	6 (13.6)	7 (20.0)	4 (8.0)	0	17 (11.9)	.179
Coronary heart disease (incidence, %)	1 (2.3)	3 (8.6)	3 (6.0)	1 (7.1)	8 (5.6)	.665

AFP = alpha-fetoprotein, CA199 = carbohydrate antigen 19-9, CEA = carcinoembryonic antigen, G-17 = gastrin-17, PGI = pepsinogen I, PGII = pepsinogen II.

* The *P*-values for comparisons between type A vs type B, type B vs type O, and type B vs type AB groups were all <.05.

### 3.2. PGI/PGII correlates

**Figure 1.**
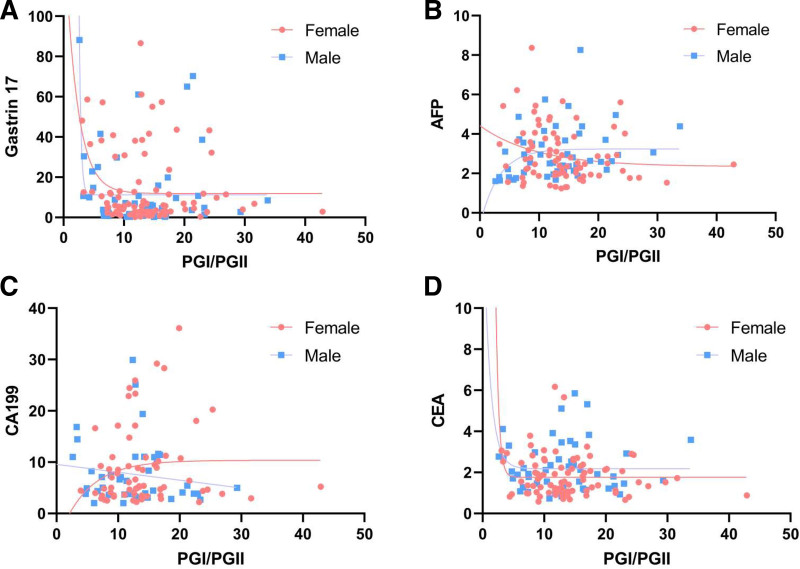
Figure displays the nonlinear curves between PGI/PGII and G-17, AFP, CEA, as well as CA199. (A) Figure demonstrates the nonlinear correlation between PGI/PGII and G-17 in both male and female groups. (B) Figure shows the nonlinear correlation between PGI/PGII and AFP in both male and female groups. (C) Figure illustrates the nonlinear correlation between PGI/PGII and CA199 in both male and female groups. (D) Figure presents the nonlinear correlation between PGI/PGII and CEA in both male and female groups. AFP = alpha-fetoprotein, CA199 = carbohydrate antigen 19-9, CEA = carcinoembryonic antigen, PGI = pepsinogen I, PGII = pepsinogen II.

Figure 1 illustrates nonlinear curves fitted via least squares algorithm to evaluate relationships between PGI/PGII ratios and biomarkers (G-17, AFP, CEA, CA199), stratified by sex. In the G-17 analysis (Fig. 1A), females exhibited a significant decline in G-17 levels at PGI/PGII ratios < 7.5, whereas males demonstrated a steeper reduction at ratios < 5. For AFP (Fig. 1B), divergent trends emerged: males showed a positive nonlinear correlation with PGI/PGII, contrasting with a negative trend in females. Notably, AFP stabilization occurred in both sexes at PGI/PGII > 10. CA199 displayed inverse sex-specific patterns (Fig. 1C), with females exhibiting a positive linear correlation and males a negative association. CEA analysis (Fig. 1D) revealed rapid declines with increasing PGI/PGII ratios across sexes, particularly pronounced in females (steeper negative slope). CEA plateaued at PGI/PGII > 5 in females.

### 3.3. Adjusted correlation heatmaps

**Figure 2.**
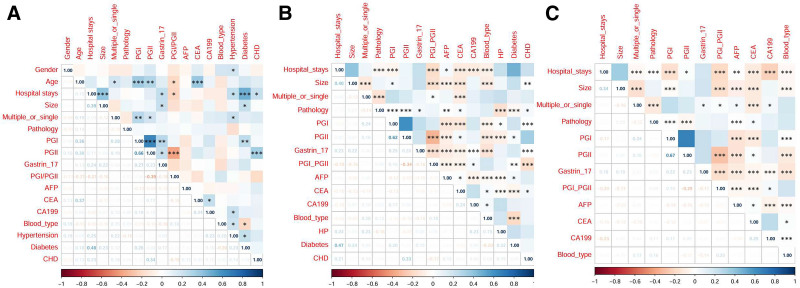
Correlation heatmaps. (A) Correlation heatmap for the entire dataset; (B) correlation heatmap after adjusting for gender and age; (C) correlation heatmap after adjusting for gender, age, hypertension, diabetes, and coronary heart disease (CHD). The correlation coefficient *r* is denoted by asterisks (*) where *, **, *** indicate statistical significance at *P* < .05, *P* < .01, and *P* < .001, respectively. Hospital stays refer to the duration of hospitalization; size refers to the size of polyps; multiple or single indicates whether the polyps are single or multiple; pathology refers to the pathological results; blood type refers to the individual’s blood group; hypertension refers to high blood pressure; diabetes refers to diabetes mellitus. AFP = Alpha-Fetoprotein, CA199 = Carbohydrate Antigen 19-9, CEA = Carcinoembryonic Antigen, CHD = coronary heart disease, gastrin-17 = gastric hormone, PGI = pepsinogen I, PGII = pepsinogen II.

Figure 2 presents the correlation analyses. The unadjusted analysis (Fig. 2A) demonstrated no significant associations between blood group and other variables. In contrast, analyses adjusted for potential confounders (Fig. 2B, C) revealed significant associations. After adjusting for sex and age (Fig. 2B), ABO blood group exhibited correlations with polyp size, PGI, PGII, G-17, and AFP. Further adjustment for hypertension, diabetes, and coronary artery disease (Fig. 2C) maintained robust associations between blood group and polyp size, PGI, PGII, G-17, AFP, CEA, and CA199, confirming the stability of these relationships.

### 3.4. Predictive value of blood group-incorporated models for gastric polyps

**Figure 3.**
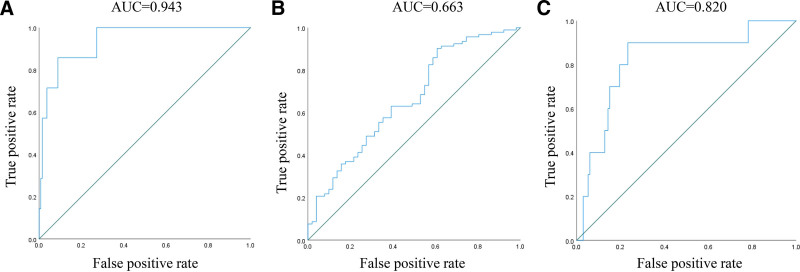
Predictive value of the model incorporating blood type for polyps. (A) Figure displays the ROC curve for predicting pathology (polyp or adenoma) using age, gastrin_17, PGI_PGII, and blood_type; (B) the ROC curve for predicting single/multiple polyps using the same variables; (C) illustrates the ROC curve for predicting polyp size (>1 cm) using age, gastrin_17, PGI_PGII, and blood_type. PGI = pepsinogen I, PGII = pepsinogen II, ROC = receiver operating characteristic.

Figure 3A demonstrates the receiver operating characteristic (ROC) curve for predicting histopathological subtypes (polyp vs adenoma) using age, sex, G-17, PGI/PGII, and blood group, yielding an area under the curve (AUC) of 0.943. For predicting polyp multiplicity (single vs multiple), the model achieved an AUC of 0.663 (Fig. 3B). The ROC analysis for size stratification (>1 cm vs ≤1 cm) showed an AUC of 0.820 (Fig. 3C).

## 4. Discussion

In recent years, while the overall incidence of gastric cancer has shown a modest decline due to increased disease awareness and dietary modifications, newly diagnosed cases are demonstrating a concerning trend toward younger age groups, posing significant threats to public health.^[16]^ Given the characteristic rapid progression and high malignancy potential of gastric cancer, early clinical intervention becomes particularly crucial.^[17]^ It is essential not to underestimate lesions with malignant potential and to implement appropriate therapeutic strategies. In the present study, we investigated the clinicopathological characteristics of gastric polyps by examining the relationships among pepsinogen levels, gastrin-17 concentrations, and ABO blood group phenotypes. Furthermore, we evaluated their predictive value for polyp multiplicity (single vs multiple) and polyp size.

Currently, gastric cancer screening modalities include endoscopy, barium radiography, and serological testing. Barium studies suffer from limited sensitivity for flat/nondepressed lesions and an inability to provide histopathological confirmation, relegating their role in modern screening paradigms.^[18]^ While EGD with biopsy represents the diagnostic gold standard, its invasive nature, procedural discomfort, and resource-intensive requirements hinder large-scale population screening.

Serological evaluation of pepsinogens (PGs) and gastrin-17 offers a noninvasive, cost-effective alternative for risk stratification. Japan and Finland have pioneered the integration of PG/G-17 panels into national gastric cancer screening protocols. Pepsinogens, 42-kDa zymogens comprising 375 amino acids, exist as 2 isoforms: PGI (exclusively secreted by gastric chief cells) and PGII (produced in pyloric/duodenal glands). Physiological PG distribution (99% luminal vs 1% systemic) confers stability to the PGI/PGII ratio, enhancing its reliability as a gastric mucosal status biomarker.^[19,20]^

Notably, this study revealed threshold effects in PGI/PGII correlations with G-17 and CEA, suggesting biological ceiling phenomena. Sexual dimorphism was observed in AFP/CA199 associations, with inverse trends between sexes. Intriguingly, ABO blood group emerged as a robust predictor after multivariate adjustment, maintaining significant associations with polyp size, PGI/PGII, G-17, and tumor markers (AFP/CEA/CA199). This underscores the necessity of incorporating demographic confounders (sex/age) in predictive modeling. ROC analyses demonstrated superior diagnostic performance for histopathological classification (AUC 0.943) and size stratification (AUC 0.820), though predictive accuracy for multiplicity remained suboptimal (AUC 0.663). Given ongoing debates regarding the clinical significance of polyp multiplicity in malignant transformation, the integration of blood group data nevertheless represents a novel advancement in personalized risk assessment.^[21,22]^

While endoscopic evaluation remains indispensable for definitive diagnosis, serological biomarkers provide a pragmatic adjunct for primary risk stratification. The synergistic integration of demographic variables (age/sex/blood group) with biochemical profiles may enhance early detection rates, ultimately contributing to reduced gastric cancer morbidity and mortality in high-risk populations.

Nevertheless, several limitations warrant consideration. First, the retrospective design inherently introduces temporal heterogeneity in disease progression that may introduce confounding factors affecting prognostic interpretation. Prospective multicenter studies with larger cohorts are required to validate these findings. Second, the pathophysiological mechanisms linking PGI/PGII, G-17, and ABO blood group phenotypes to gastric polyp malignant transformation remain incompletely elucidated. Third, the single-center recruitment strategy carries inherent selection bias, potentially limiting generalizability. External validation through multi-institutional cohorts is imperative for clinical translation.

In conclusion, this study demonstrates the substantial clinical utility of a multiparametric model integrating age, sex, PGI/PGII ratio, G-17 levels, and ABO blood group phenotypes in stratifying histopathological subtypes, polyp multiplicity, and lesion dimensions among patients with gastric polyps. These findings advocate for the incorporation of serological and demographic biomarkers into risk stratification algorithms to optimize diagnostic precision and therapeutic decision-making.

## Author contributions

**Investigation:** Jun Tang.

**Writing – original draft:** Qian Gao.

**Writing – review & editing:** Xing He, Haiqing Li, Hezhong Yan, Lili Wu, Pei Zhong.

## Supplementary Material


