# Supplementary figures and images for: Serological and genetic markers in gastric polyps: Diagnostic and prognostic roles of pepsinogen, gastrin-17, and ABO antigens
Source: Medicine (Baltimore). 2026 Jan 23;105(4):e46964. doi: 10.1097/MD.0000000000046964 (PMC12851752; doi:10.1097/MD.0000000000046964)

**Supplementary Fig.** The flow chart of this study.

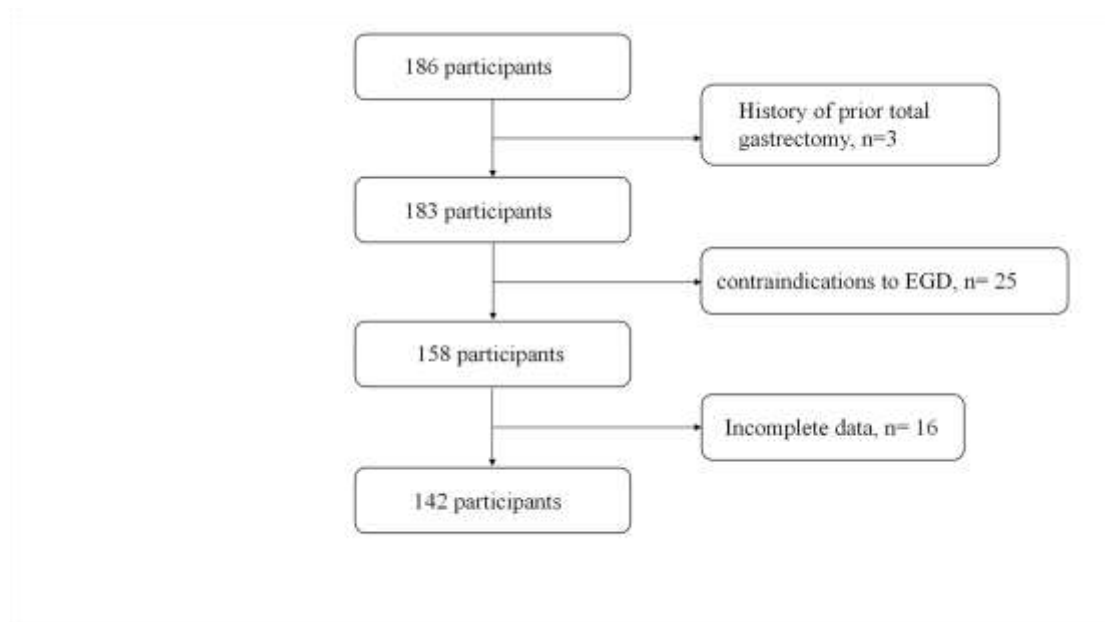

Supplement: Supplementary file 1 [file medi-105-e46964-s001.pdf]
